# Dr. Lubbock's Observations on the Discoveries of Mayow

**Published:** 1799-05

**Authors:** Richard Lubbock


### *To the Editors of the Medical and Phyfical Journal.*

GENTLEMEN,

I Submit to your confideration the following obfervations upon the difcoveries of MAYOW; a fubject, which has, lately, employed the pens, and attracted the attention of different literary characters, in this ifland, and on the continent.

I am, Gentlemen, your obedient fervant,

RICHARD LUBBOCK.

Much and deferved praife is due to the zeal and exertions of Dr. BEDDOES, and Dr. YEATS, in their attempts to render juftice to the writings and difcoveries of Mayow, by calling the attention of the learned to the merits of this great philofopher, the KEPLER, perhaps the NEWTON, of chemical fcience.

It appears however to me, from the perufal of fome contemporary and fubfequent writers, found, chiefly, in a private and provincial collection of books, that the knowledge of Mayow's writings was, as much, and as generally, diffufed over Europe, immediately upon, or very foon after their publication, as of any work of fcience ever publifhed, in which the march and progrefs of the human mind was equally outftript, by equal depth of refearch or boldnefs of difcovery; and the advocates of Mayow have, perhaps, been too rapid in their conclufions, in afferting, that contemporary writers received his works with an ungrateful filence, or ignorant infenfibility.

In

In ſupport of the early attention of the learned to Mayow's writings, it may be remarked, that the preſs at Oxford, in the courſe of a few years, ſent forth two editions of ſome, if not of all his treatiſes; and that the 'Tractatus quinque' were alſo very ſoon reprinted in Holland. It may alſo be obſerved, that MANGETUS was not inſenſible of the value of his writings, having, about the end of the laſt century, reprinted his treatiſes on Reſpiration, and on the Fœtus in utero, in his "*Bibliotheca Anatomica*", and having quoted them, in terms of praiſe, in other parts of the ſame work.

It is with ſome ſurprize, that I find Dr. Yeats giving it as his opinion, that MUNDY, in his work, printed at the ſame time with Mayow's "*De Aëre Vitali, &c.*", and alſo reprinted in Holland, in 1684, has not mentioned the name of Mayow, nor adopted his opinions; when, at the end of the ſeventh chapter, "*De Atmoſpheræ pondere, et Aëris elatere*", he is quoted in the following terms: "Alioquin (inquit D. J. Mayow, qui de his rebus ingenioſe commentatus eſt) nulla omnino foret hominum, imo ne animalium quidem ſocietas; quippe opporteret, ut ſinguli et ſeparatim vitam degerent, quo, viz. ſpiritus aëris penus ad vitam ſuſtinendam ſtis amplus, cuique ſuppeteret".—The other parts of the ſame chapter amply prove, that Mundy embraced the opinions of Mayow, upon the nature of air. There was alſo printed at Oxford, in 1694, a work, entitled, "*Diſſertationes Medico-Phyſicæ, de Antris Lethiferis, etc.*". by Dr. CONNOR, in which, the name of Mayow is duly quoted, and the general reception, which his doctrine had met with, is candidly acknowledged, and in the following words, "Objicietur procul dubio, quod in Antris ferialibus, animantia et faces nullatenus ex aëris, ſed ex nitri aëris defectu expirant.—Receptiſſima enim eſt ubique gentium opiniode nitri aëris efficacia; qua contenditur poſt Cl. D. Mayow, quod aëris nitrum in ſanguinem introducitur per pulmones"; and after ſome ſimilar obſervations, Dr. Connor concludes, "et arrogantiæ reus viderer, ſi ſolus ego tot præclaros viros, qui hanc opinionem utriſque ulnis amplexi ſunt, et foverunt, a veritatis tramite deviaſſe aſſererem"

In 1694, Dr. Coward alſo publiſhed his work, de "Fermento volatili nutritio," in which Mayow's work is referred to more than once; and to the above names, I cannot forbear adding, (with Dr. Yeats,) that of the excellent Collins, whoſe anatomy was printed in 1685. Thus it appears, that Mayow's Diſcoveries attracted, and in no ſmall degree, the attention of contemporary writers in this kingdom.

Nor were they leſs known on the Continent; for Peyer in his "Parerga anatomica et emedica," (Amſterdam, 1682,) quotes and approves of Mayow's opinions; and in 1692, he is quoted, in a very ingenious work, publiſhed a at Geneva, "De prima coctione & fermento ventriculi", by Viridetus—and Etmuller examines his opinions, largely, in the edition of his works, printed 1697, in his Treatiſe on Reſpiration; and it muſt not be forgotten, that about, or ſoon after this period, Mayow's opinions were referred to by ſome of the firſt philoſophers and medical characters of the age. It will be ſufficient to ſay, that John Bernouilli quotes Mayow, in his diſſertation, "De motu muſculari, and de fermentatione,"--that the learned Pitcairn quotes him, in his "Opuſcula medica"; that Morgagni notices his opinions, in his "Adverſaria"; and that Bianchi, in his orations, at the end of his 'Hiſtoria hepatica,' mentions him, among other diſcoverers in phyſiology. But I muſt conclude, by remarking, that Mayow's opinions are frequently cited, in the "Exercitationes medicæ", printed at London, in 1724, by John Tabor, and who has mentioned his works in the following manner: Mirifici hujus ſalis præconia, a nullo vel plenius, vel magis ex profeſſo celebrantur, quam a noſtrate Mayow; e cujus ſcriptis tanquam e promptuario, haud pauca argumenta fuere deſumpta, nitro aëreo plurimum referta; quæ, in oſtendendis aëris muneribus ad ignis accenſionem, vegetabilium productionem, atque ad animalium motus omnes vitales edendos, totam rem confeciſſe credita ſunt."

It muſt be here admitted, that the fore-mentioned authors have quoted Mayow, for the moſt part, on Phyſiological ſubjects, ſuch as on the function of the ſtomach, and of the lungs, and upon muſcular motion; and that ſome of them muſt be conſidered ſolely as friendly to the extention of his name, and not as advocates for the truth of his doctrines. And the reaſon that his chemical philoſophy has been leſs noticed, is, that the opinions of Stahl were very ſoon made public, appealing, for their ſupport, to a warm imagination, rather than exerciſing the judgment by the labour of induction, or accurate inveſtigation.

Norwich, April 16th,

1799.

