# Errata

**Published:** 1799-07

**Authors:** 


					ERRATA.
jSo. hi. p. 221, 1. 24, for " antris fenalibus," read c antris ferahbus f

				

